# Zebrafish Models to Study New Pathways in Tauopathies

**DOI:** 10.3390/ijms22094626

**Published:** 2021-04-28

**Authors:** Clément Barbereau, Nicolas Cubedo, Tangui Maurice, Mireille Rossel

**Affiliations:** MMDN, Univ Montpellier, EPHE, INSERM, 34095 Montpellier, France; clement.barbereau@laposte.net (C.B.); nicolas.cubedo@umontpellier.fr (N.C.); tangui.maurice@umontpellier.fr (T.M.)

**Keywords:** tauopathies, zebrafish, frontotemporal lobar degeneration, Tau protein, Tau hyperphosphorylation)

## Abstract

Tauopathies represent a vast family of neurodegenerative diseases, the most well-known of which is Alzheimer’s disease. The symptoms observed in patients include cognitive deficits and locomotor problems and can lead ultimately to dementia. The common point found in all these pathologies is the accumulation in neural and/or glial cells of abnormal forms of Tau protein, leading to its aggregation and neurofibrillary tangles. Zebrafish transgenic models have been generated with different overexpression strategies of human Tau protein. These transgenic lines have made it possible to highlight Tau interacting factors or factors which may limit the neurotoxicity induced by mutations and hyperphosphorylation of the Tau protein in neurons. Several studies have tested neuroprotective pharmacological approaches. On few-days-old larvae, modulation of various signaling or degradation pathways reversed the deleterious effects of Tau mutations, mainly hTauP301L and hTauA152T. Live imaging and live tracking techniques as well as behavioral follow-up enable the analysis of the wide range of Tau-related phenotypes from synaptic loss to cognitive functional consequences.

## 1. Introduction

Tauopathies are neurodegenerative diseases that all share a common spread of neurotoxicity associated with pathological intracellular deposits of Tau proteins.

The term tauopathy was proposed in 1997 by Spillantini and includes diseases that combine Tau intracellular deposition, which can occurs in neurones, glia, or oligodendrocytes and involved ultimately cell degeneration [1]. Tauopathies can present clinical features ranging from dyskinesis and movement disorders to dementia. Today, about 20 tauopathies have been identified, the most known of which is Alzheimer’s disease [2]. We will focus on a large group of tauopathies: frontotemporal lobar degeneration (FTLD), which includes frontotemporal dementia linked to chromosome 17 (FTDP-17), progressive supranuclear palsy (PSP), cortico-basal degeneration (CBD), argyrophilic grain disease (AgD), or Pick’s disease.

Due to their number and diversity, neuropathologists determine specific characteristics in order to classify these tauopathies [3].

## 2. Multi-Criteria Classification of Tauopathies

Tauopathies can be classified into two groups: primary tauopathies are characterized by the presence of solely Tau aggregates in brain tissue and secondary tauopathies include on the other hand, other protein aggregates in association with Tau cellular deposits, such as Aβ amyloid in Alzheimer’s disease, for example. Within primary tauopathies, the cell type can also be considered together with Tau intracellular aggregates, as defining criteria. Thus, the age-related tau astrogliopathy, ARTAG group has been characterized by Tau deposits in glial cells, as described for some PSP [4].

From a genetic point of view, hereditary tauopathies are characterized by the presence of a variant in the MAPT gene encoding the Tau protein or inheritance of the H1 haplotype of the MAPT locus [5]. Variants in the MAPT gene were found in both coding and non-coding sequences in FTDP-MAPT [6]. Many of these mutations are localized in the microtubule binding domains and the C-terminal part of the Tau protein. In this group Tau deposit morphology includes neurofibrillary tangles (NFTs).

NFTs are also in primary age related tauopathy group (PART) as well as other diseases such as some PSP [7]. PART is a group of diseases primarily affecting neurons, which have NFT in brain areas similar to those found in Alzheimer’s disease [5,8,9,10].

**Figure 1:**
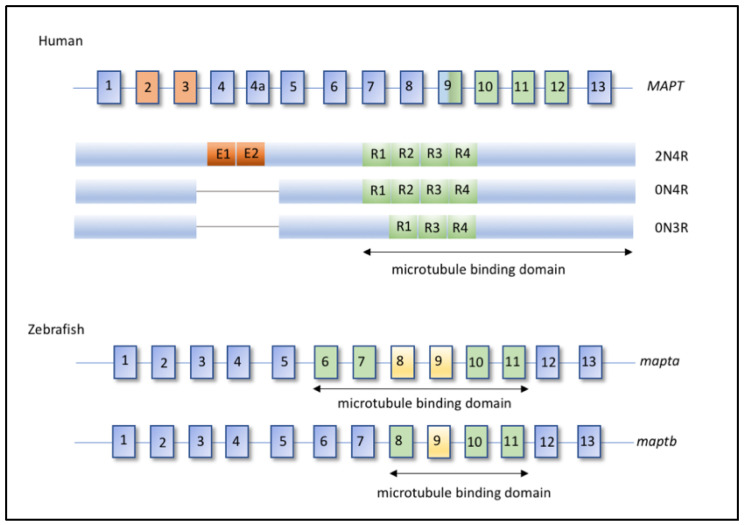
Organization of the human and zebrafish *MAPT* genes. Human *MAPT* gene is represented with the major isoforms used in transgenic models and found in patients, 2N4R, 0N4R, and 0N3R. Orange exons encode N-terminal domain and green exons encode repeats domain (R) that belong to microtubule binding domains. Exon 10 is alternatively spliced to generate 3R isoform. Zebrafish *mapta* and *maptb* genes have 13 exons each (blue), with green exons encoding microtubule binding domains, and yellow one labelling alternatively spliced exons.

Finally, another biochemical classification considers the isoforms of the Tau protein present in Tau aggregates: the 3R/4R ratio (Table 1, Figure 1). Some tauopathies have been shown to involve equally the 3R and 4R isoforms of the Tau protein, including Alzheimer’s disease and some FTDP-17 [11]. Tauopathies characterized by a majority or exclusivity of Tau protein isoform 4R, ratio ranging from 1/2 to 1/4, encompass some types of PSP, CBD or AgD [12]. In the particular case for Pick’s disease, a higher level of Tau protein isoform 3R compared to 4R is observed [13] (Table 1).

These different classifications of tauopathies highlight the complexity and diversity of this group of diseases. Thus, the majority of the described diseases may combine several criteria.

PSP is classified as primary tauopathy with or without mutation in the *MAPT* gene. Aggregates can be found in neurons (NFTs or filaments) as well as glial cells but only the Tau 4R isoform is involved. On the contrary, FTDP-17 are primary tauopathies with different mutations in the *MAPT* gene. They may have a single specific pathological isoform (Tau3R or Tau4R) or both. All these tauopathies lead to brain atrophy linked to degeneration and neuronal death. However, clinical signs change depending on the type of disease (Table 1) [5,14].

## 3. Tau Protein Dysfunctions: Hyperphosphorylation and Aggregation

Tau belongs to the MAP (microtubule associated protein) family whose main function is to ensure the stability of the microtubule network. Tau is encoding by the *MAPT* gene, which undergoes alternative splicing resulting in 6 isoforms. These isoforms include, or not, a N-terminal insert and 3 or 4 microtubule- binding domains (Figure 1), resulting in 3R or 4R forms [15]. Tau has also been described at the level of the plasma membrane [16] as well as in the nucleus of neurons [17] suggesting a complex protein with multiple roles. Moreover, Tau is submitted to numerous post-translational modifications, which may influence its subcellular localization or modulate its affinity for microtubules [18]. In adult neurons, the main isoforms are 3R/4R with an equimolar ratio. When involved in pathologies, Tau proteins are found associated in the form of oligomers, aggregates and possibly into neurofibrillary tangles (NFT) which are associated with cell degeneration. Tau proteins found in these different forms carry a large number of post-translational modifications that induce significant changes in the function of the Tau protein, and can lead to a toxic and pro-aggregative gain of function.

Phosphorylation is the main post-translational modification commonly found in NFT Tau species. Under physiological conditions, it has been determined that 2 to 3 phosphates are carried by Tau protein, which appears to be a normal phosphorylation of the protein. Under pathological conditions, the number of phosphate groups per Tau protein increases drastically. It has been determined that hyperphosphorylation can occur at 7 to 8 phosphates per Tau protein [19].

In all tauopathies, aggregated Tau proteins are found to be hyperphosphorylated. In patients with tauopathies, and more particularly with Alzheimer’s disease, some domains, such as the proline-rich and the C-terminal regions, contain a large number of phospho-rylated sites [20]. Abnormal phosphorylation of the Tau protein has a major role in Tau-related pathology development.

NFT formation is a progressive process that seems to be linked to different post-translational modifications. The last stage of NFTs corresponds to an accumulation of Tau proteins aggregates, organized in paired-helical filaments in the cell. These NFTs are composed of an overwhelming majority of Tau proteins, amputated from their terminal ends, making them even more insoluble [21].

## 4. Zebrafish Humanized Lines to Study Tauopathy

Tauopathies are complex and multifactorial diseases, and need appropriate models to understand pathology development. Current mouse models partially reproduce the major deficits (amnesia and neurotoxicity) found in humans due to specific promotor driven human *MAPT* mutated gene. Moreover, these mouse models associate one or several mutations of *MAPT*, to obtain the late onset of deficits [22,23,24,25]. It is therefore important to develop scalable systems that allow the study of several factors to be combined concomitantly or sequentially. Zebrafish is an alternative of choice that allows the development of neurotoxicity induced by the deregulation of the expression of candidate proteins in just a few days. Indeed, this vertebrate model completes its embryonic development in two days and reaches the stage of autonomous larva at 5 days. Because of its transparency at embryonic and larval stages, many reporter lines with fluorescent and/or photoactivatable proteins have allowed to follow the development of the nervous system as well as the development of neurotoxicity.

Therefore, zebrafish has emerged as a complementary model to study neurodegenerative diseases compared to rodent, invertebrate and cell lines. Due to evolutionary conserved gene function, neurodegenerative process and pathways can be recapitulated in zebrafish model. Using genome editing and/or human gene expression as knock-in or transient expression strategies, gene function involved in disease development can be followed in embryo and larvae that allow live imaging of neuronal process.

Due to genome duplication in zebrafish, two genes homologous to the human MAPT gene encoding the Tau protein have been identified: *mapta* and *maptb* [26] (Figure 1). From 24 h post fertilization (hpf), both mRNAs are detected throughout the central nervous system of the zebrafish [26]. Alternative splicing of the different RNAs of the *mapta* and *maptb* genes show that different isoforms are present. The *mapta* RNAs can contain between 4 and 6 microtubule-binding domains and the *maptb* RNAs have 3 microtubule-binding domains. These isoforms are strongly reminiscent of the 3R and 4R isoforms found in humans (Figure 1).

Several transgenic, transient or stable lines have been developed to study different tauopathies. To restrict the expression of the transgene to the nervous system, different promoters were used. The first report of Tau transgenic zebrafish was provided by Tomasiewicz in 2002. In their study, the 4R isoform of the human Tau protein fused with eGFP under the control of the promoter *Gata-2* is transiently expressed [27]. This *Gata-2* promoter, strongly activated during development, allows expression of the transgene restricted to the central nervous system [28]. At 48 hpf, 10 to 15% of neurons underwent destabilization of the microtubule network and accumulation of the Tau protein in the cellular body. These proteins are phosphorylated at S396 and S404 sites and appear to assemble in neurofibrillary tangles [27].

**Figure 2:**
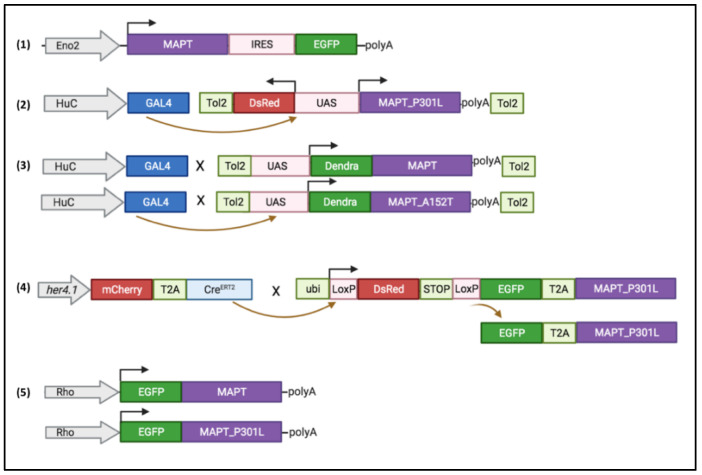
Transgenes used to generate stable lines. (**1**) Human *MAPT* 4R isoform is expressed under Eno2 promoter [29]. (**2**) Double transgenic line using Gal4/UAS system: *Gal4* is expressed under *HuC* promoter and bind to bidirectional *UAS* promoter to induced the expression in the same neuron of DsRed and hTauP301L proteins [30]. (**3**) Using *HuC* promoter driving *Gal4* line cross with UAS-driven fused *Dendra:MAPT* or *Dendra:MAPTA152T* results in neuronal expression of human proteins Dendra-hTauWT or Dendra-hTauAT52T [31]. (**4**) Conditional transgene using CreERT2_LoxP recombination: tamoxifen incubation results in expression of 2 proteins: EGFP and hTauP301L in her4.1 positive neural stem cells [32]. (**5**) Retina expression of *MAPT* or *MAPTP301L* driven by rhodopsin (Rho) promoter [33,34].

A first stable transgenic line was conducted by Bai and his team in 2007 and expressed a 4R isoform of the human Tau protein under the control of the *Eno-2* promoter (Figure 2, #1). The *Eno-2* promoter induces a neuronal expression. While this promoter is weakly activated during development (up to 72 hpf), its activation increases until adulthood [29]. This line leads to widespread expression of the Tau protein in the optic tectum and the thalamus. A refractile pattern was observed for neuronal somatic Tau immunolabelling, which suggest fibrillar deposition however no Gallyas staining was performed.

Other transient expression studies have been conducted by Wu and co-workers: they induced overexpression using plasmids carrying the coding sequences for the zebrafish Tau 3R or human protein Tau 4R fused to GFP under the control of a HuC promoter. The HuC promoter drives early neuronal expression during zebrafish development [35]. Wild type and truncated forms prone to aggregate in vitro were studied in parallel. All these Tau protein isoforms cause an increase in cell death and an increase in phosphorylation of the Tau protein at 24 hpf. Treatments with different neuroprotective factors, such as Bcl2, Nrf2, or GDNF, showed a rescue of neuronal death without decreasing the phosphorylation found on Tau protein at 48 hpf [36].

### 4.1. Zebrafish hTauP301L Model

**Figure 3:**
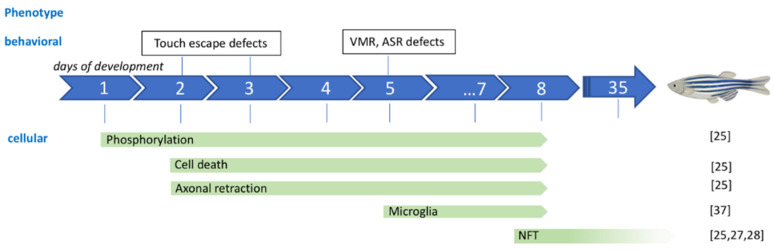
Phenotype evolution during zebrafish development for the hTauP301L mutation. NFT: neurofibrillary tangles; VMR: visual motor response, ASR: acoustic startle response.

The first stable transgenic line expressing a mutated human Tau protein was produced by Paquet and his team in 2009 (Figure 2, #2). This line carries two transgenes: a transgene containing the coding sequence for the human Tau protein carrying the P301L mutation and the *DsRed* reporter gene as well as a second transgene containing the coding sequence of the *Gal4* transcription factor, under control of the *HuC* promoter [30]. The transcription of both the *hTauP301L* and *DsRed* genes is under the control of a bidirectional *UAS* promoter, which induces transgene transcription after binding of the Gal4 protein. This system provides neuronal expression of the *hTauP301L* and *DsRed* transgenes within the same cell (Figure 2). Expression of the *hTauP301L* transgene in this lineage leads to neuronal and behavioral changes within 48 hpf with an altered locomotor response for the escape reflex (touch escape response). Thus, an increase of the phosphorylation of the Tau protein, at the level of epitopes recognized by AT8 (Ser202 and Thr205) and Phf1 (Ser396 and Ser404) considered pathological, was found as early as 48 hpf for this line. At 6- and 7-days post-fertilization (dpf), an increase in neuronal death and a decrease in axonal projections were detected for this hTauP301L line. At five weeks, the transgenic line shows positive aggregates to the Gallyas stain at the level of the spinal cord, which would correspond to neurofibrillary tangles. Finally, using Gsk3β kinase inhibitors, it appears that phosphorylation of the Tau protein on Serine 396 and Serine 404 is decreased [30] (Figure 3).

A conditional model was created by Cosacak et al., with expression of the 2N/4R isoform of the human Tau protein carrying the P301L mutation under tamoxifen induced Cre recombination. In this transgenic line, hTau expression is under the control of the *her4.1* promoter that allows expression of the transgene in stem cells from the embryonic stage. Phenotypes have been evaluated on the larva and on the zebrafish brain in adulthood. While Tau protein hyperphosphorylation was observed, no oligomer nor neurofibrillary tangles were detected. No increase in cell death by apoptosis or microglial activation was observed and no behavioral deficits could be identified. Injection of Aβ42 peptides into this line did not show an aggravation of phenotypes compared to Aβ42 peptide-induced phenotypes alone or hTauP301L alone [32].

Besides transgenic lines that overexpressed hTau in nervous system, tissue specific expression was obtained with stable expression under the zebrafish rhodopsin promoter in the retina of fish larvae (*Rho:EGFP-Hsa.MAPT* and *Rho:EGFP-HsaMAPT_P301L*) [33,34]. Photoreceptor specific expression of hTau lead to protein accumulation and degeneration with neurofibrillary tangles in the photoreceptor layer as early as 8dpf. (Figure 3).

### 4.2. Zebrafish hTauA152T Model

Lopez and colleagues generated two other lines that expressed a wild type human Tau protein or the hTauA152T variant, associated with higher risk of FTDP and PSP. These lines express a Tau 2N4R protein fused with the Dendra protein as a reporter. The transgene expression is driven by *UAS* promoter which is activated by the Gal4 protein under the control of the *HuC* promoter (Figure 2, #3). Lopez et al. analyzed in parallel hTauA152T and hTauWT control lines. The expression of the hTauA152T transgene in larvae causes, from 3 dpf, important morphological defects, with a marked curvature of the larvae body [31].

In addition, motor neurons have axonal projections with abnormal orientations and aberrant ramifications in hTauA152T larvae. An increase in the phosphorylation of the Tau protein is found for hTauA152T and particularly in the pathological epitopes AT8 (Ser202 and Thr205) and Phf1 (Ser396 and Ser404) at 24 hpf. This increase in phosphorylation of the Tau protein is related to the increase in neuronal death at 3 dpf. In addition, at the behavioral level, a decrease in the flee response to the touch escape response test was observed for hTauA152T at 6 dpf. The authors highlighted a decline in proteasome activity, however, the degradation pathway through autophagy did not appear to be affected. An increase in autophagy showed beneficial effects for hTauA152T, with improved larval morphological defects, responses to behavioral tests and decreased phosphorylation of the Tau protein. According to this study, the activation of autophagy pathway therefore seems to be a candidate of interest to repair the deficits found in tauopathies.

## 5. What Do We Learn from These Models?

New altered pathways have been highlighted through live observations and pharmacological approaches.

Using hTauP301L line, several groups identified potential neuroprotective factors or Tau interacting protein such as FKBP52, an immunophilin protein that interacts with physiological Tau through tubulin binding domain. Giustiniani et al., identify a direct interaction between FKBP52 and hTauP301L phosphorylated forms and its ability to induce the formation of Tau-P301L oligomers [37]. Neurotoxicity mechanisms can also be deciphered, using for example live imaging of microglia behavior during disease progression. Hassan-Abdi et al. showed that in presence of diseased neurons, microglia exhibited a higher mobility and displayed morphological changes, with fewer and shorter processes. In addition, genetic ablation of microglia in zebrafish hTauP301L line significantly increased Tau hyperphosphorylation, suggesting that microglia provide neuroprotection to diseased neurons [38]. Mitochondrial behavior defect was also observed from live record of axonal mitochondrial transport. Mitochondria seemed to move faster with a trend toward longer pauses between runs resulting in a compensation of the accelerated moving speed [39].

A new interacting candidate was validated in hTauP301L line, the Heparan sulfate (glucosamine) 3-O-sulphotransferase 2, hs3st2. This compound participates to the molecular mechanisms leading to abnormal phosphorylation of Tau. Its pharmacological inhibition results in a strong reduction of the aberrant phosphorylated Tau epitopes in brain and in spinal cord. Therefore hs3st2 inhibitors lead to a complete recovery of motor neuronal axons length and functional rescue of the animal motor response to touch-evoked response stimuli [40]. In line with these results, Alavi Naini et al. have applied surfen or oxalyl surfen treatments on hTauP301L larvae and obtained an improvement on the neurotoxicity phenotype. Surfen might therefore modulate Tau phosphorylation through heparan sulfate biosynthesis [41].

Recently, hTauP301L larvae analysis revealed a decrease of the neurotrophic factor BDNF while its TrkB receptor was not affected. BDNF rescue attempt was successful to prevent the locomotor phenotype observed for this line at 5dpf, i.e., the hyperactivity response after visual motor stimulation. In addition, the agonist 7,8 DHF compound for the TrkB receptor, completely rescued the visual motor response of hTauP301L larvae [42]. As Tau neurotoxicity is linked to accumulation of abnormal forms of Tau, hyperphosphorylated and/or oligomer, a strategy of elimination of these toxic forms through the enhancement of 26S proteasome could be beneficial. Such hypothesis has been tested on the rhodopsin-Tau lines and Huc-Dendra-TauA152T (#3 and 5, Figure 2). Interestingly, agents that activate the proteasome via cGMP, such as phosphodiesterase (PDE) inhibitors, sildenafil, PDE5 inhibitor, or rolipram, PDE4 inhibitor, rescue Tau-induced photoreceptor cell death and morphological abnormalities [34]. Thus, the increase of Tau degradation in vivo prevents aggregates formation and results in protective action.

Overexpression of stable human WT or mutated Tau proteins in zebrafish neurons lead in most cases to neurotoxicity but not exactly to the pathological hallmarks as in human syndromes. Interestingly, different mutation types give rise to common defects with hyperphosphorylation of Tau protein and cell death reflecting Tau-dependent toxicity, mimicking deficits observed in human. However NFTs are rarely observed, only in long term follow-up [30] or in retina [33]. The majority of these studies analyze phenotypes induced by the expression of a wild or mutated human Tau protein from embryonic to larval stages. They allow a rapid study of neurotoxicity, behavioral defects and deregulated pathways by overexpression of this protein, with pharmacological approaches to test new neuroprotective compounds. Models studying the effect of the Tau protein on adult stages should provide an opportunity to explore other systems, such as memory, social behaviors, or conditioned responses [43,44]. It would be interesting to develop new lines under the control of a specific promoter allowing a high expression of Tau transgenes in adult zebrafish leading to Tau aggregate formation up to pre-tangles and/or neurofibrillary tangles which are rarely retrieved in this organism. This point might be highly interesting to decipher and to understand how zebrafish neural cells can control Tau intracellular deposits.

## 6. Neurosensory and Locomotor Tests Developed in Zebrafish

Pharmacological approaches to prevent or rescue Tau-dependent phenotypes in larvae have been successfully applied as early as 24 h of development up to 5 days. Given the wide range of pathway tested, more signalization pathways have been uncovered and candidate neuroprotective factors validated using screening methods with 96 well plates functional tests. Using neurobehavioral platforms, several exploratory methods have already yielded promising results on measuring for example hearing loss on zebrafish larvae modeling neurodegenerative diseases, and identifying molecules currently in Phase I clinical trials in humans [45,46]. Numerous other studies use larval zebrafish behavior or locomotion in an effort to identify pharmacological molecules with a beneficial effect on Tau pathologies [47].

Zebrafish larvae acquire functional sensory and locomotor systems early on. For example, as early as 18 hpf, spontaneous contractions of the larva from tail to head can be observed. By 2 dpf, the larva is performing short, fast, straight swims that will evolve into continuous swims by 3 dpf [48]. At 2 dpf, the mechanosensory system of zebrafish larvae is also operational which allows for the “touch escape response” test. By 4 dpf, the olfactory and visual systems of the larvae, despite the fact that they are not yet fully differentiated, are able to respond to stimuli [49,50]. At 5 dpf, the auditory system of larval zebrafish is also able to perceive sound stimuli [51] as well as habituation to repeated light or sound stimuli [52]. Finally, at 10 dpf, zebrafish larvae show the first social behaviors, visualized with the shoaling test [53].

**Figure 4:**
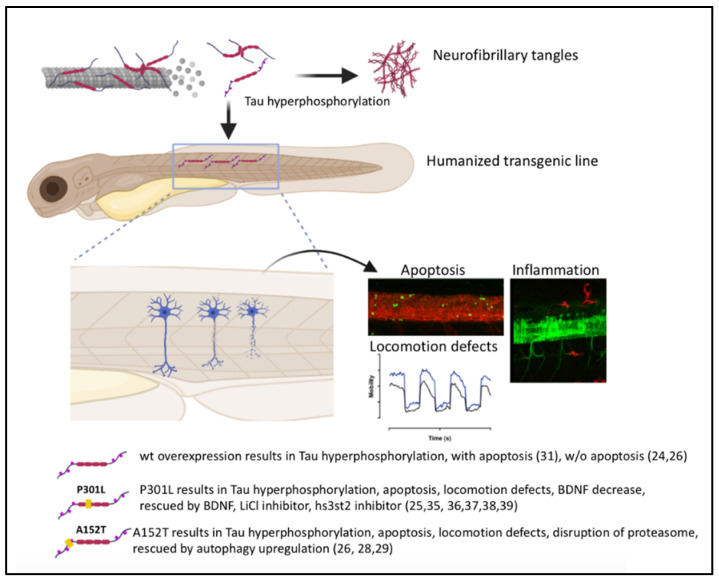
Summary of the main strategies and findings in Tauopathy zebrafish models. Upon hyperphosphorylation, Tau protein detached from the microtubules and form oligomers. Oligomer accumulation led to pre-tangles and in some cases to neurofibrillary tangles. Zebrafish transgenic lines overexpressed human Tau proteins in neurons which led to apoptosis (green cells), inflammation response with microglia activation (red cells) and locomotion defects (visual motor response profile). Transgenic lines for human Tau WT or P301L and A152T mutations, revealed that neuroprotective factors can rescue neurotoxicity as well as activation of the proteasome and autophagy pathways.

Therefore, behavioral tests can be performed early on to analyze the motor performance as well as the integrity of the sensory systems of zebrafish larvae. The “touch escape response” induces a locomotor reflex after gentle touch stimulation. To test the locomotor activity related to the presence or absence of light, the visual motor response (VMR) test is based on alternating periods with light ON and OFF. The typical profile response of 5 dpf larvae is a very low locomotor activity in the presence of light, and an increased locomotor activity in the dark (Figure 4). These VMR involve both visual system and locomotor activity [54,55]. In addition, the oculomotor and visual capacities, can be analyzed with the optokinetic response (OKR). The OKR test consists in scrolling black and white vertical stripes in front of immobilized larvae. The larvae will respond with eye saccades which correspond to the tracking of the strips [45,56,57] The auditory system is tested using the acoustic startle response (ASR) that induced a locomotor response following a short sound stimulation. However, sound stimulation also induces a vibration in the water which can potentially trigger a response related to the lateral line mechano-sensory system [58,59].

Due to the high capacity of regeneration of the zebrafish tissues, the full spectrum of phenotypes should be analyzed. Even if hTauP301L has been the most studied mutation in zebrafish but also in mouse, more hTau mutation model are being developed in order to decipher Tau mutation-dependent mechanisms. For example, in the case of PSP pathology, whose symptoms range from Parkinson-like problems to dementia and aphasia, it is necessary to study in vivo the etiology of neurotoxicity. Sensory functions could be included in the spectrum of Tau phenotypes such as visual, auditory and balance anomalies. As retina cell death has been observed in hTau152T larvae, functional defect might be uncovered, using visual tests such as visual motor stimulation and optokinetic responses.

## 7. Concluding Remarks

The models generated to study these tauopathies have made it possible to show deregulated signaling pathways by the transgenic expression of a human Tau protein. Several of them, representing potential neuroprotective pathways, have been explored through pharmacological approaches. As mentioned above, tauopathies include frequently astrogliopathy, which has not been studied so far. In order to provide a closer model to human symptoms, zebrafish will allow the combination of several *GAL4/UAS* transgenes with specific promoter overexpression as for example *HuC* (neurons) and *GFAP* (astrocytes) in the same embryos. Using such models in zebrafish will contribute to understand how glial cells, astrocytes and/or oligodendrocytes, are involved to the development of tau pathology.

## Figures and Tables

**Table 1:** Molecular pathological classification of tauopathies.

Tauopathies	AD	CBD	Pick’s Disease	FTDP-MAPT	PSP	AGR
*MAPT* mutation	-	-	+	+	+/-	-
Isoform ratio 3R/4R	3R = 4R	3R < 4R	3R > 4R	3R < 4R	3R < 4R	3R < 4R
Tau phosphorylation	+	+	+	+	+	+
Tau aggregates: neurons (N) or glia (G)	N	N,G	N	N,G	N,G	N,G

AD: Alzheimer’s disease, AGR: argyrophilic grain disease, CBD: cortico-basal degeneration, FTDP: frontotemporal dementia linked to *MAPT* mutation, PSP: progressive supranuclear palsy [4,14].

## Data Availability

Not applicable.
